# Comprehensive analysis of the biological functions of endoplasmic reticulum stress in prostate cancer

**DOI:** 10.3389/fendo.2023.1090277

**Published:** 2023-03-10

**Authors:** Shengren Cen, Dongmei Jiang, Daojun Lv, Ran Xu, Jiamao Hou, Zixiang Yang, Peng Wu, Xinhao Xiong, Xingcheng Gao

**Affiliations:** ^1^ Department of Urology, The First Affiliated Hospital of Guangzhou Medical University, Guangzhou, China; ^2^ Department of Pathology, The First Affiliated Hospital of Guangzhou Medical University, Guangzhou, China; ^3^ Department of Urology, The Third Affiliated Hospital of Guangzhou Medical University, Guangzhou, China

**Keywords:** prostate cancer, endoplasmic reticulum stress, BCR, immune environment, drug sensitivity

## Abstract

**Introduction:**

Endoplasmic reticulum stress (ERS) has sizeable affect on cancer proliferation, metastasis, immunotherapy and chemoradiotherapy resistance. However, the effect of ERS on the biochemical recurrence (BCR) of prostate cancer patients remains elusive. Here, we generated an ERS-related genes risk signature to evaluate the physiological function of ERS in PCa with BCR.

**Methods:**

We collected the ERS-related genes from the GeneCards. The edgeR package was used to screen the differential ERS-related genes in PCa from TCGA datasets. ERS-related gene risk signature was then established using LASSO and multivariate Cox regression models and validated by GEO data sets. Nomogram was developed to assess BCR-free survival possibility. Meanwhile, the correlations between ERS-related signature, gene mutations, drug sensitivity and tumor microenvironment were also investigated.

**Results:**

We obtained an ERS risk signature consisting of five genes (AFP, COL10A1, DNAJB1, EGF and PTGS2). Kaplan Meier survival analysis and ROC Curve analysis indicated that the high risk score of ERS-related gene signature was associated with poor BCR-free prognosis in PCa patients. Besides, immune cell infiltration and immune checkpoint expression levels differed between high- and low-risk scoring subgroups. Moreover, drug sensitivity analyzed indicated that high-risk score group may be involved in apoptosis pathway.

**Discussion:**

This study comprehensively analyzed the characteristics of ERS related genes in PCa, and created a five-gene signature, which could effectively predict the BCR time of PCa patients. Targeting ERS related genes and pathways may provide potential guidance for the treatment of PCa.

## 1 Introduction

Prostate cancer (PCa) is the most prevalent tumor in the male reproductive system. The estimated number of new cases of PCa diagnosed in 2022 is 268,490, with a 6% annual increase in the incidence of distant-stage disease since 2011 (1). To make matters worse, the 5-year survival rate for those cases with distant metastases dropped dramatically, to almost 30% (2). For localized PCa, radical prostatectomy and radiotherapy are the recommended interventions, and although this treatment strategy can benefit a large amount of patients with PCa, some patients are still at risk for biochemical recurrence (BCR) (3, 4). Therefore, a better understanding of the BCR of PCa may contribute to effective early diagnosis and targeted therapy. In the present study, we pay attention to the biological function and prognostic value of PCa endoplasmic reticulum stress (ERS).

ERS is an imbalance in endoplasmic reticulum (ER) homeostasis caused by the accumulation of unfolded or misfolded proteins and changes in Ca^2+^ concentration (5). The normal function of ER requires a stable cellular microenvironment, and the dysfunction of ER has an important effect on various cellular processes (6). More than 30% of all proteins made in the cell required ER for synthesis, folding, and structural maturation (7). Plenty of studies have shown that ERS participated in the occurrence and development of many human malignancies (8). ERS has also been reported to play a crucial role in the proliferation and apoptosis of cancer cells in a hypoxic environment and has been associated with resistance to radiotherapy and chemotherapy (9, 10). In PCa, IRE1α, PERK, and ATF6H are activated when cellular stress is detected in the ER to trigger unfolded protein responses leading to survival-friendly effects (11), suggesting a critical function of ERS in PCa progression. However, the critical functions of ER stress and its downstream signaling pathways in PCa progression are not well understood and still deserve further clarification. A comprehensive investigation of ERS may help to develop a sound PCa diagnosis and treatment strategy.

Currently, we have collected the sequencing and clinical data of PCa from The Cancer Genome Atlas (TCGA) and obtained ERS-related genes from GeneCards. Next, we calculated the differential expression of ERS-related genes between PCa tissue and paracancerous tissue. Based on these genes, we divided patients into two groups using the ConsensusClusterPlus package. Then, we developed a five-ERS-related-gene signature by least absolute shrinkage and selection operator (LASSO) and Cox regression to evaluate BCR-free prognosis of PCa patients in the TCGA and Gene Expression Omnibus (GEO) datasets. We also constructed a nomogram to predict the BCR possibility using risk score and other related clinical parameters. Furthermore, we divided the patients into two subgroups based on the risk score of the ERS signature and found significant differences in the level of immune cell infiltration, somatic mutations, expression level of immune checkpoints, and drug responses between the two risk groups. In conclusion, these results provide evidence that ERS signaling is critical for the progression of PCa, and elucidating the function of ERS signaling may provide new insights into the treatment of PCa.

## 2 Materials and methods

### 2.1 Data collection and processing

The transcriptome profiling and clinical data of PCa were obtained from TCGA (https://portal.gdc.cancer.gov) with TCGAbiolinks (HTSeq-Counts). Two other datasets (GSE21034 and GSE70770) were accessed from GEO (https://www.ncbi.nlm.nih.gov/geo/). The TCGA-PRAD dataset was selected by the following steps: (1) The follow-up was more than 20 days. (2) Samples without complete BCR follow-up clinical information were removed. We obtained 411 PCa patients with complete BCR follow-up information and 406 patients with complete clinical information (**Table S1**). The EdgeR package was used to analyze differentially expressed genes (DEGs) between tumor and paracancerous tissue on the R 4.1.3 platform, and FDR < 0.05 and |log2-fold change| ≥ 1 were considered to be statistically significant. ERS-associated genes were collected from GeneCards (https://www.genecards.org/), and the correlation score ≥ 7 was selected, as Huang et al. reported (12).

### 2.2 Functional enrichment analysis

Gene Ontology (GO), Kyoto Encyclopedia of Genes and Genomes (KEGG), and gene set enrichment analysis (GSEA) were used to perform enrichment analysis of the DEGs with the “ClusterProfiler” package (13). Gene Set Variation Analysis was carried out with “GSVA” packages.

### 2.3 Consensus clustering analysis of ERS-related genes

The ConsensusClusterPlus package (14) was used to perform unsupervised hierarchical clustering to identify differentially expressed ERS-related clusters *via* pam algorithms. A total of 1,000 iterations were carried out to ensure the stability of these categories. The “proportion of ambiguous clustering” (PAC) was applied to infer the optimal number of clusters, where the *K* value has the lowest PAC.

### 2.4 Development of the ERS-associated BCR prognostic signature

The common genes in TCGA-PRAD DEGs and ERS-related genes were selected for univariate Cox regression analysis. Then, these BCR-related genes were retained for the LASSO model with the glmnet package, and 10-fold cross-validation was accepted to select the minimal penalty term. Then, the remaining genes were used to establish an optimal ER stress-related risk model, using the Akaike information criterion (AIC) method of multivariate Cox regression analysis. The ERS signature risk score was calculated as follows: risk_score = **∑*_i_*_=1_ *^n^*** (Coefi × Expi), where Coefi is the corresponding regression coefficient evaluated by multivariate Cox regression model and Expi is the expression value of the ERS-related genes. We divided patients into high-risk and low-risk groups based on the median risk score. Additionally, the TCGA-PRAD cohort was used as a training set to evaluate the prognostic value of BCR-dependent receiver operating characteristic (ROC) curves at 1, 3, and 5 years; GSE21034 and GSE70770 were utilized for the validation cohorts.

### 2.5 Drug sensitivity prediction

Drug-response prediction was evaluated based on the V2 database (809 cell lines and 198 compounds) of the Genomics of Drug Sensitivity in Cancer (GDSC) (15), using the “oncoPredict” package (16), and the half-maximum inhibitory concentration (IC_50_) of each patient was assessed using the Ridge Regression model.

### 2.6 Mutation analysis of the risk score model

The R package “TCGAmutations” was used to calculate the total somatic mutation of TCGA-PRAD between different risk score subgroups. The online tool Sangerbox (17) (http://vip.sangerbox.com) was adopted to map an oncoplot to show the mutation landscape in high-risk and low-risk groups.

### 2.7 Establishment of a predictive nomogram

Based on the TCGA-PRAD dataset, a nomogram was established to predict the 1-, 3-, and 5-year BCR prognosis of PCa patients with ERS-related risk score and other related clinical parameters, using the “rms” package. Decision curve analysis and C-index were used to validate the clinical reliability of the nomogram model.

### 2.8 BCR prognostic validation of risk score

Univariate Cox and multivariate Cox regression were performed with risk score and other clinical variables to identify whether the ERS score was an independent prognostic predictor. The correlation of ERS-related genes’ risk score and age, Gleason score, and T stage were calculated.

### 2.9 Tumor immune microenvironment in PRAD

The ESTIMATE package was used to evaluate the immune and stromal scores of PCa. The MCPcounter and CIBERSORT packages were used to detect the infiltration level of 22 immune cells and two stromal cells. Additionally, we also analyzed the tumor immune dysfunction and exclusion (TIDE) and MSI score for PCa patients in the two groups *via* TIDE tool (http://tide.dfci.harvard.edu). The immune checkpoints SIGLEC15, CTLA4, CD274, IDO1, PDCD1, HAVCR2, PDCD1LG2, and LAG3 between two groups were also analyzed.

### 2.10 Statistical analyses

The bioinformatics analysis was performed with R 4.1.3 (https://www.r-project.org/). Mann–Whitney–Wilcoxon tests or Student *t*-tests were carried out to analyze continuous variables. Survival plots were calculated by a log-rank test using the “survival” packages. The drug response was tested with Spearman’s correlation analysis. We chose *p* < 0.05 as the statistical significance.

## 3 Results

### 3.1 Exploration of endoplasmic reticulum stress-related genes

**Figure 1:**
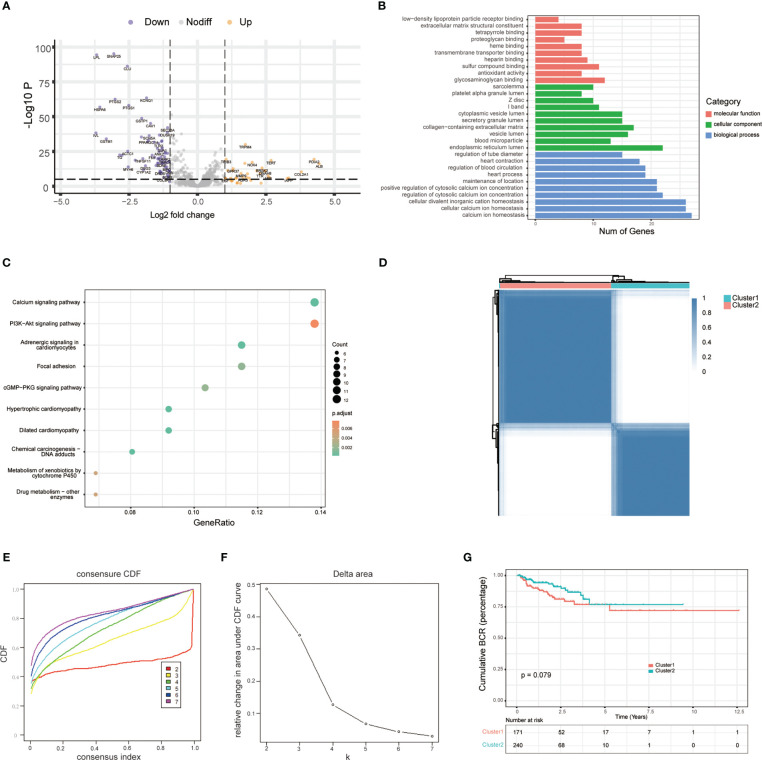
Exploration of endoplasmic reticulum stress-related genes. **(A)** Volcano plot shows differentially expressed endoplasmic reticulum stress-related genes between tumor and adjacent normal tissue in the TCGA database. **(B)** GO analysis terms of reticulum stress-related DEGs. **(C)** The top 10 most enriched KEGG pathways of ERS-related DEGs. **(D)** Consensus matrices of the TCGA-PRAD cohort for *k* = 2. **(E)** Consensus values range from 0 to 1. **(F)** The corresponding area under the cumulative distribution function (CDF) curve changes relatively as the number of clusters changes from *k* to *k* + 1. *k* ranges from 2 to 7, optimal *k* = 2. **(G)** Survival analysis of patients between cluster 1 and cluster 2.

To characterize the role of ERS-related genes, we extracted 787 ERS-related genes with correlation scores ≥7 from the GeneCards, as reported by Zhang et al. (12). We then analyzed the DEGs of these ERS-related genes in the TCGA cohort. As shown in **Figure 1A**, we obtain 108 DEGs. GO and KEGG enrichment analysis suggested that these DEGs were mainly enriched in the calcium ion homeostasis, calcium signaling pathway, glycosaminoglycan binding, PI3K-Akt signaling pathway, and ER lumen and adrenergic signaling in cardiomyocytes (**Figures 1B, C**). Additionally, based on the ERS-related DEGs, we classified PCa patients into two clusters with consensus clustering (**Figure 1D**). The cumulative density function and PAC method were used to identify the optimal *k* value (**Figures 1E, F**). We further performed survival analysis on these two clusters and found that the trend of BCR survival was worse in cluster 1 than in cluster 2 (**Figure 1G**), but not statistically significant, probably because of the number of queues, which requires further study.

### 3.2 Development of five endoplasmic reticulum stress-related gene risk signature

**Figure 2:**
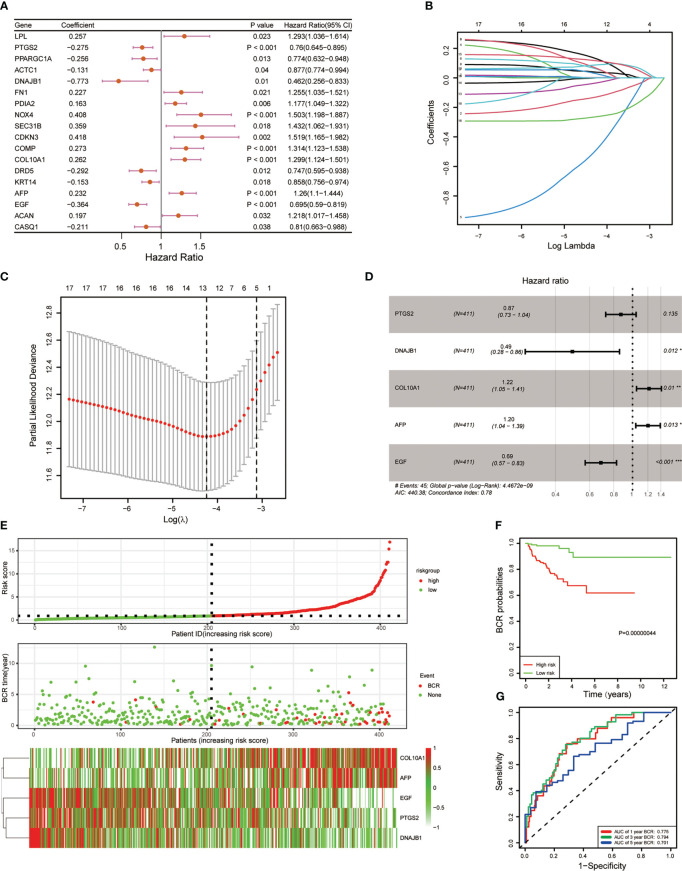
Development of five endoplasmic reticulum stress-related gene BCR signature. **(A)** Univariate Cox regression revealed 18 ERS-related genes associated with BCR. **(B)** Eighteen ERS-related genes were penalized by LASSO Cox regression analysis. **(C)** Tenfold cross-validation for the optimal parameter selection in the LASSO Cox regression. **(D)** A five-gene model was constructed with a stepwise regression model using the Akaike Information Criterion (AIC) method. **(E)** The risk score distribution, BCR status, and five-gene expression trend in the TCGA PRAD dataset. **(F)** KM survival curve of the five-gene signature in the TCGA PRAD dataset. **(G)** ROC curves for BCR-free survival prediction models at 1, 3, and 5 years. (*p < 0.05; **p < 0.01; ***p < 0.001).

To further construct the BCR prognostic model, we performed univariate Cox regression on ERS-related DEGs and identified 18 genes that were associated with BCR prognosis (**Figure 2A**). Furthermore, we analyzed these 18 genes by LASSO regression and derived 5 genes based on the minimum partial likelihood deviation (**Figures 2B, C**). Next, a five-gene BCR prognostic risk model was constructed by multivariate Cox regression analysis based on the AIC value (**Figure 2D**). Subsequently, the PCa cohort was divided into two subgroups based on the median risk score according to the risk model, and the expression of these five genes was shown in the heatmap (**Figure 2E**). BCR-free survival was analyzed using Kaplan–Meier and log-rank tests, and the results showed that the high-risk group had a shorter BCR-free time than the low-risk group (**Figure 2F**). In addition, the ROC curve was applied to assess the predictive efficiency of the model. The area under the ROC curve (AUC) was 0.775, 0.794, and 0.701 for 1, 3, and 5 years, respectively (**Figure 2G**), which indicated that the model performed well in predicting BCR-free survival in the TCGA cohort.

### 3.3 Validation of the ERS-related gene model with the external dataset

**Figure 3:**
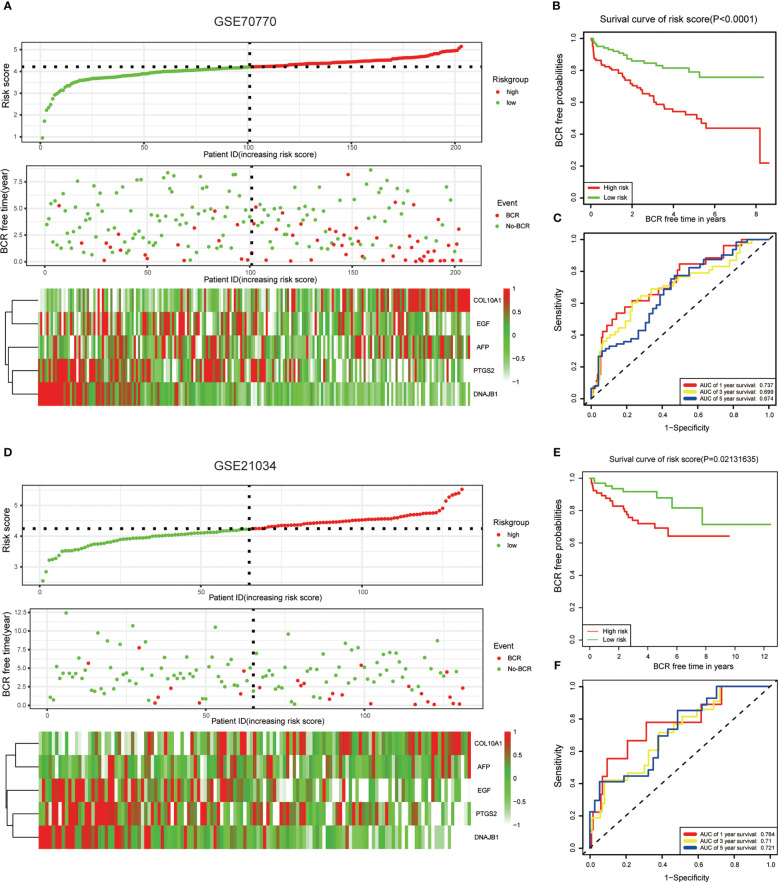
Validation of the ERS-related genes model with the external dataset. **(A, D)** The risk score distribution, BCR status, and five-gene expression trend in GSE70770 and GSE21034. **(B, E)** KM survival curve of the five-gene signature model in GSE7070. **(C, F)** ROC curves for BCR-free survival prediction models at 1, 3, and 5 years. The risk score in both datasets was calculated by the same formula and divided into high-risk and low-risk groups based on the median risk score.

To better evaluate the predictive efficiency of the ERS-related gene model, we selected GEO datasets GSE70770 and GSE21034 for further validation. Risk scores were calculated for all patients in both datasets according to the same formula. We then divided the two datasets into high-risk and low-risk groups according to the median risk score. The edgeR package was then accepted to evaluate the expression patterns of these five genes in two GEO datasets and was found to be similar to TCGA (**Figures 3A, D**). As expected, survival time without BCR was significantly shorter in the high-risk group compared to the low-risk group (**Figures 3B, E**). Furthermore, we detected the predictive efficiency of this five-gene risk model utilizing ROC curves in GSE70770 and GSE21034, and the AUCs of 1, 3, and 5 years in both datasets suggested that the model was stable (**Figures 3C, F**).

### 3.4 Integrated analysis of risk models and clinical characteristics

**Figure 4:**
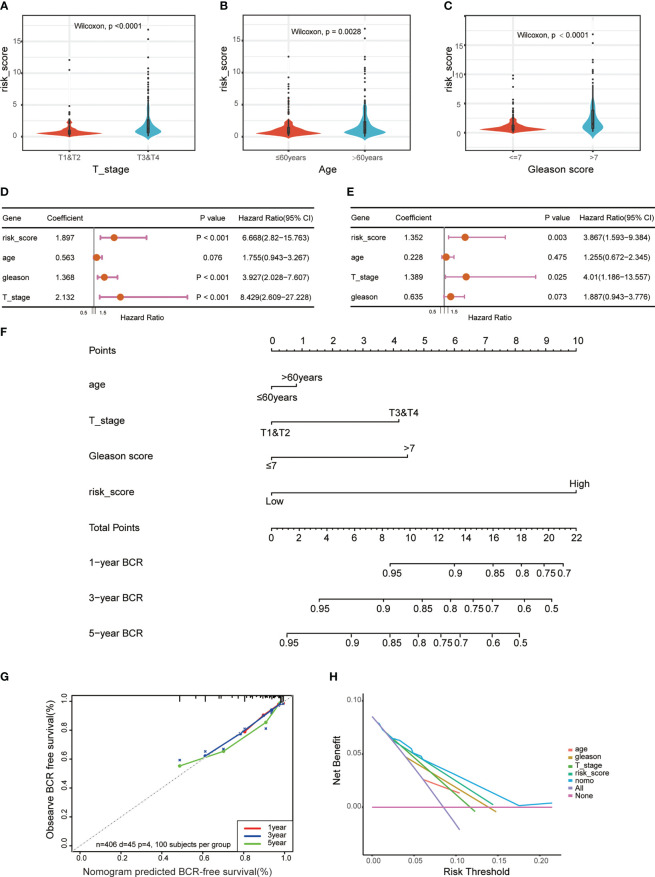
Correlations between risk models and clinical characteristics based on the TCGA PRAD dataset. Violin plot shows different ERS risk score between different pathological stage **(A)**, age **(B)**, and Gleason score **(C)** of the TCGA PRAD cohort. Univariate **(D)** and multivariate **(E)** Cox regression analyses were used to explore the prediction of ERS-associated risk signature in the TCGA PCa dataset. **(F)** A nomogram with ERS-related risk scores, age, T stage, and Gleason scores for predicting the probability of BCR-free survival in patients. **(G)** The calibration curves of the nomogram. **(H)** The decision curve analysis (DCA) for median BCR-free survival time prediction.

To investigate the function of this ERS-related gene risk model in the clinical characteristics of PCa, we assessed the association between risk scores and clinical features. Results showed that compared with the low-risk group, the high-risk group had a higher tumor Gleason score and a more aggressive tumor stage (**Figures 4A, C**). The age of high-risk cohort patients was higher than that of the low-risk group (**Figure 4B**). In addition, univariate and multivariate Cox regression analysis suggested that the risk score was an independent prognostic factor in the TCGA dataset (**Figures 4D, E**) and extra GEO cohorts (**Figures S1D–G**). To better predict the BCR-free survival of PCa, we constructed a nomogram with ERS-related genes’ risk scores, age, tumor stage, and Gleason scores (**Figure 4F**). ROC curves were used to estimate the performance of nomogram, age, risk score, and tumor stage in predicting 1-, 3-, and 5-year BCR-free survival (**Figures S1A–C**). The calibration curves were utilized to assess the predictive performance of this nomogram with respect to the actual observed BCR-free survival rate (**Figure 4G**). The DCA curves indicated that the ERS-related gene risk signature had a favorable predictive efficiency (**Figure 4H**).

### 3.5 Functional enrichment analysis between different risk types

**Figure 5:**
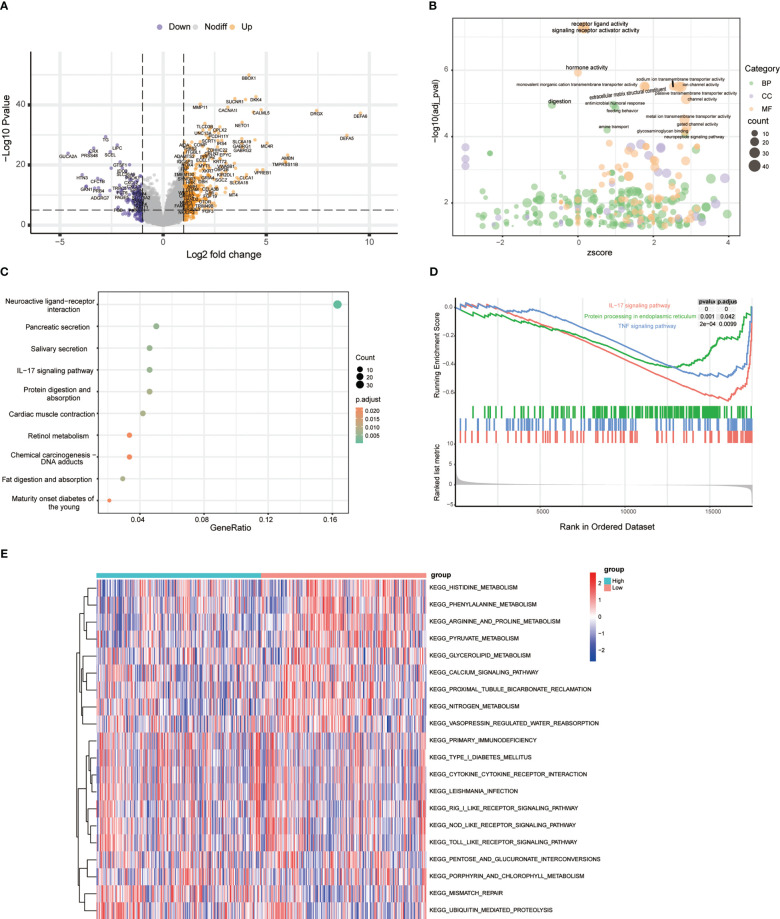
Functional enrichment analysis between risk types. **(A)** Volcano plot of differentially expressed genes between high- and low-risk groups. **(B)** Bubble chart showing GO terms of differentially expressed genes between different risk types. **(C)** Top 10 KEGG pathways of differentially expressed genes between different risk types. **(D)** GSEA results between high-risk and low-risk groups. **(E)** The heatmap shows the top 10 upregulated and top 10 downregulated GSVA scores for KEGG pathways grouped by high- and low-risk group.

To identify the underlying mechanisms in different risk groups, we analyzed the DEGs in different risk subgroups in the TCGA cohort, and obtained 547 DEGs (|logFC| ≥ 1, *p*-value < 0.05) (**Figure 5A**). GO enrichment analysis was performed to assess the function of these DEGs, and the result showed that they were mainly involved in signaling receptor activator activity, receptor ligand activity, and hormone activity (**Figure 5B**). In addition, KEGG and GSEA showed that these ERS-related DEGs were mainly enriched in the IL-17 signaling pathway, ER protein processing, and TNF signaling pathway (**Figures 5C, D**). Furthermore, gene set variation analysis showed that the high-risk group was enriched in inflammatory and immune-related pathways, such as primary immunodeficiency and the Nod-like receptor signal pathway. Pathways related to histidine metabolism, phenylalanine metabolism, and calcium signaling were also varied in two groups (**Figure 5E**). The above results show that these DEGs participate in various processes including immune activity.

### 3.6 Integrated analysis of ERS risk signature and immune cell infiltration in PCa

**Figure 6:**
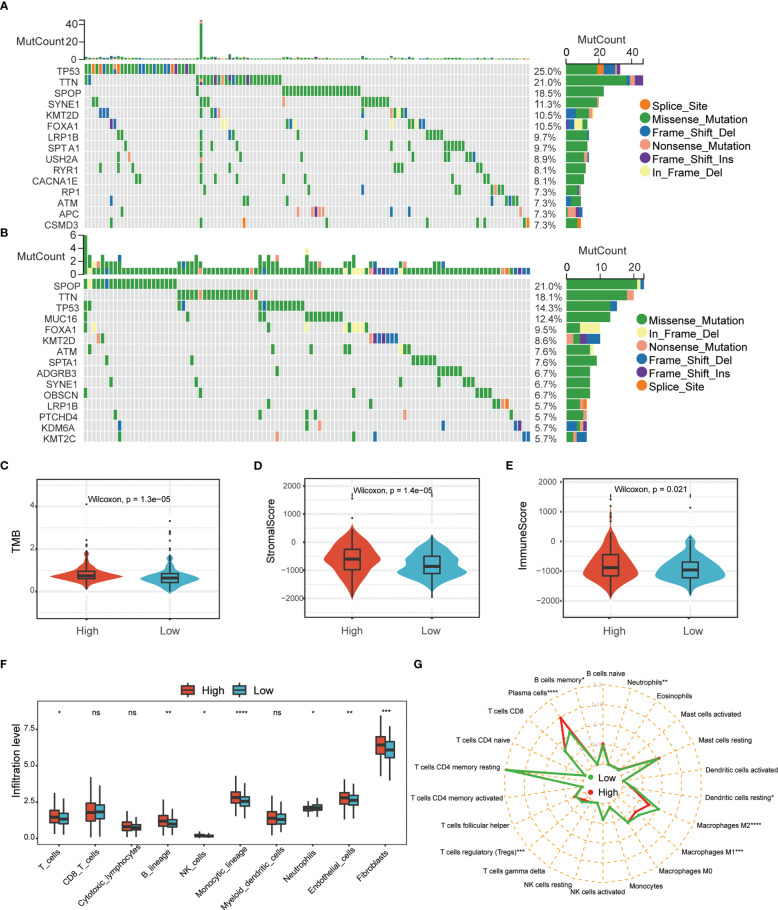
Comprehensive analysis of ERS-associated signature score and immune cell infiltration in PRAD. Waterfall plot of the top 15 somatic mutation signatures for groups with high **(A)** and low **(B)** ERS risk scores. **(C)** Relationships between ERS risk score and tumor mutational burden (TMB). Correlations between ERS risk score and both stromal **(D)** and immune scores **(E)**. MCPcounter **(F)** and CIBERSORT **(G)** were used to analyze the degree of immune cell infiltration in the two groups (ns, not significant; *p < 0.05; **p < 0.01; ***p < 0.001; ****p < 0.001).

To further understand the potential characteristics of these two populations, we analyzed the top 15 individual cell mutation genes in both the high- and low-risk groups. TP53, TTN, SPOP, SYNE1, and KMT2D were the top five genes with the highest mutation frequency in the high-risk group, while in the low-risk group, they were SPOP, TTN, TP53, MUC16, and FOXA1 (**Figures 6A, B**). Meanwhile, patients in the high-risk group had higher levels of total mutational burden (TMB) than those in the low-risk group (**Figure 6C**). These results suggested that ERS-related genes may act through genetic mutations. Gene mutations can generate new antigens, and we wanted to know if the expression of immune checkpoints and immune infiltrating cells differed in the two tumor immune environments. We uncovered that PCa in the high-risk group had higher stromal and immune score (**Figures 6D, E**). MCPcounter showed that the high-risk group PCa had a higher abundance of T cells, NK cells, monocyte lines, and neutrophils (**Figure 6F**). CIBERSORT suggested that resting dendritic cells, Tregs, and macrophages M1 and M2 were significantly enriched in the low-risk group (**Figures 6G**, **S2**). TIMER analysis showed that DNAJB1, COL10A1, PTGS2, AFP, and EGF were correlated with tumor-infiltrating lymphocytes (**Figure S3**). Taken together, these data indicated that the different BCR-free prognosis may be related to the infiltrating immune cells.

### 3.7 Relationship between ERS risk score and immune response and drug sensitivity

**Figure 7:**
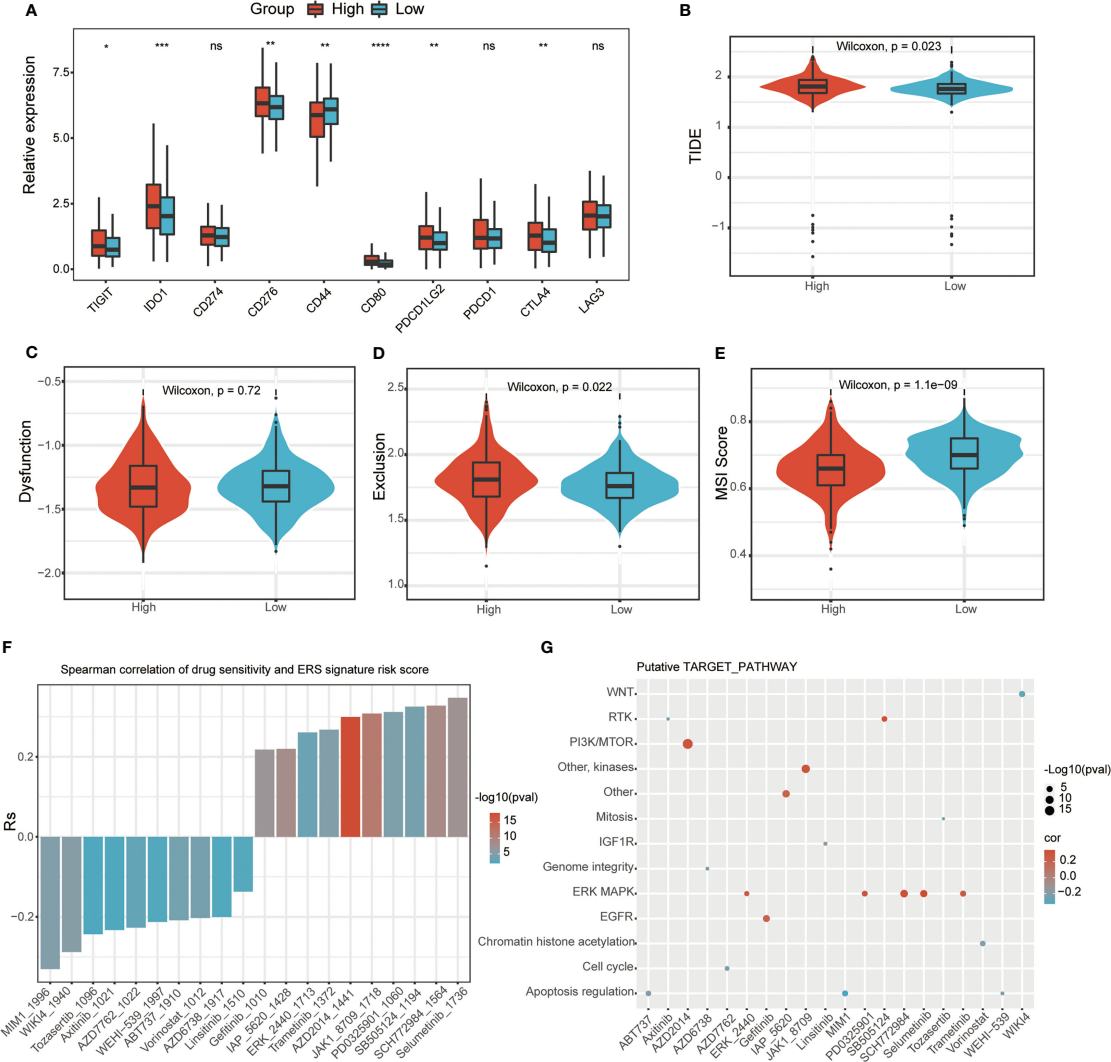
Relationship between ERS-related signature scores and immune response and drug sensitivity. **(A)** Immune checkpoint analysis between two ERS risk groups (ns, not significant; *p < 0.05; **p < 0.01; ***p < 0.001; ****p < 0.001). TIDE **(B)**, dysfunction score **(C)**, and T-cell exclusion **(D)** and MSI **(E)** in different ERS risk groups in the PRAD dataset. **(F)** The correlation between ERS risk scores and drug sensitivity (AUC values of GDSC) examined by the Spearman analysis. **(G)** Putative targets or functional pathways of the drugs that are sensitive to the ERG risk scores (ns, not significant; *p < 0.05; **p < 0.01; ***p < 0.001; ****p < 0.001).

Since the current study indicates that ERS-associated signature scores are associated with immune cell infiltration, we sought to find a link between ERS-associated gene risk scores and immune responses. This study evaluated the immune checkpoints in different subgroups based on risk score. The results showed that the expression levels of many immune checkpoints were lower in the low-risk subgroup than in the high-risk subgroup (**Figure 7A**). Moreover, the current study calculated TIDE scores between these two risk types, and the data showed that the low-risk group had a higher MSI score but a lower TIDE score and a lower T-cell dysfunction score (**Figures 7B–E**), indicating that the T-cell immune checkpoint inhibitor may be more effective in patients with a low-risk score. Furthermore, based on the GDSC database, the current study uses the “oncoPredict” package to look for compounds that may interact with ERS-related pathways. The results showed that ERS risk score-related drug sensitivity was related to apoptosis regulation (MIM1, WEHI-539, and ABT737), cell cycle (AZD7762), and WNT (WIKI4). ERS risk score-related drug resistance was associated with ERK MAPK (Selumetinib, SCH772984 and PD0325901), PI3K/MTOR (AZD2014), and EGFR (Gefitinib) (**Figures 7F, G**).

## 4 Discussion

PCa has been ranked first in incidence and second in estimated mortality among men (18). Although PCa progresses relatively slowly in its early stages compared to other cancers, approximately 35% of patients will experience BCR within 10 years of radical prostatectomy (19). BCR is characterized by a continuous postoperative PSA ≥ 0.2 ng/ml and is universally accepted for monitoring the prognosis of PCa patients (20). When PCa undergoes BCR, it usually becomes more aggressive, even metastatic, and life-threatening, especially if the Gleason score is high (21, 22). Therefore, there is great clinical value in identifying appropriate biomarker signatures to predict early BCR after radical prostatectomy. Clinicopathological features, such as the clinical stage (T), Gleason score, and PSA, are the key risk factors for BCR after radical prostatectomy (4, 23). Over the past decade, ERS has become an increasingly compelling area of research for various human cancers (24), which could become a new strategy for the therapy of PCa.

The ER is an important organelle, known for protein synthesis and intracellular calcium storage, and is involved in various cellular signaling pathways, such as lipid biogenesis, calcium metabolism, and autophagy signaling pathways (5, 25, 26). Chronic ERS is considered to be the key pathophysiological cause of cell damage in many popular human diseases, including diabetes, neurodegenerative diseases, stroke, and cancer (27, 28). Excessive and sustained activation of ERS interferes with ER function, leading to accumulation and aggregation of unfolded proteins, which then activate JNK and other apoptosis-related signaling pathways, leading to cell death (29, 30). Although the ERS has been reported to have a vital function in PCa progression, there was lack of integrated analysis of ERS-related genes in BCR of PCa, and the understanding of ERS may increase the choice of cancer therapy and improve the prognosis of PCa patients.

ERS-related genes and PCa transcriptome data were obtained from public databases, and then the ERS-related gene risk signature was constructed and validated using the TCGA and GEO datasets. We divided patients into high-risk (above the median) and low-risk groups (below the median) based on the median ERS-related gene signature risk score. Prognostic analysis showed that the high-risk group had a shorter BCR-free survival time. Moreover, we constructed a nomogram model, which proved to have a favorable prognostic performance. These results suggest that the ERS-related gene risk score is an independent BCR-free prognostic factor for PCa patients. Furthermore, among these five genes, DNAJB1, EGF, and PTGS2 were positively associated with BCR-free time survival, while COL10A1 and AFP showed the opposite effect. DNAJB1 has been reported to be a cancer biomarker for targeted therapy and prognosis of pancreatic cancer (31). EGF has been reported to be associated with aggressiveness and progression-free interval in PCa patients treat with androgen blockade (32). PTGS2 DNA fragment in the serum of PCa patients could be used as a diagnostic and prognostic marker (33). COL10A1 from cancer-associated fibroblasts promotes LUSC cell proliferation and inhibits oxidative stress-induced apoptosis, and may also serve as a potential biomarker for gastric cancer progression and prognosis (34, 35). In addition, AFP is a popular clinical biomarker for HCC, and it can also be used as a potential prognostic biomarker for PCa (36, 37).

Furthermore, we performed an enrichment analysis of DEGs for both risk types and found that these genes were mainly focused on metabolic and immunoregulatory pathways, such as the IL17 signaling pathway, fat and protein digestion and absorption signaling pathway, and TNF signaling pathway. All these signaling pathways had an important function in tumor progression. IL-17 signaling has been reported to induce translation of HIF1 α, which then drives immune exclusion by activating the collagen deposition program in murine models of cutaneous squamous cell carcinoma (38). Activation of the TNF-α/TNFR2 axis can promote the immunosuppressive phenotype and function of Tregs, leading to cancer progression (39, 40). Moreover, we assessed the relationship between the ERS signature risk scores and the TMB and immune microenvironment of PCa and found that the TMB was higher in the high-risk group than in the low-risk group. The gene with the highest mutation frequency in low-risk patients was SPOP, while TP53 was in the high-risk group. It is well accepted that somatic mutations are the cause of cancer and are associated with the production of neoantigens (41, 42). Increased infiltrating immune cells and mutational burden are highly correlated with prognosis and may serve as predictors of cancer immunotherapy (43). Here, we found that the high-risk subgroup had higher levels of immune checkpoints and a relatively active immune cell infiltration compared to the low-risk group. However, the TIDE analysis predicted that the low-risk group may respond well to immunotherapy. The detailed mechanism needs further exploration. Finally, the association between ERS risk scores and drug response was also assessed. The results implied that the drugs sensitive to ERS-related high-risk scores targeted apoptosis regulation, cell cycle, and WNT signaling pathways, whereas those in the low-risk score group mainly targeted ERK MAPK, PI3K/MTOR, and EGFR signaling pathways.

Taken together, all data suggest that ERS is involved in the progression of PCa. On the basis of ERS-related genes, we developed a prognostic model for BCR, and with the help of ERS risk signature, we could adjust the treatment of patients to some extent. However, our study also has several unavoidable limitations. First, biochemical-based endpoints may not be suitable as a proxy for meaningful survival outcomes in PCa, and the association between ERS-related genes and distant metastasis-related outcomes was not well analyzed. Second, the function of ERS associated with BCR-free survival has not been confirmed by our own data cohort. Future large-scale prospective studies and molecular experiments are needed to validate these findings.

## 5 Conclusion

We constructed a five-gene risk signature based the ERS-related genes to evaluate the role of ERS in PCa patients. The BCR prognosis, somatic mutation, infiltration levels of immune cell, and drug response were different between the two risk groups. By integrating ERS signature risk scores and clinical parameters, we further constructed a nomogram, further demonstrating its good predictive performance. Potential therapeutic compounds targeting ERS were also evaluated. These results may provide new insights into the identification of prognostic biomarkers and the development of therapeutic targets.

## Data availability statement

Publicly available datasets were analyzed in this study. This data can be found here: The Cancer Genome Atlas (TCGA) (https://portal.gdc.cancer.gov) and https://www.ncbi.nlm.nih.gov/geo/ (GSE21034 and GSE70770).

## Ethics statement

The study was reviewed and approved by the ethics committee of the First Affiliated Hospital of Guangzhou Medical University (Guangzhou, China). Written informed consent for participation was not required for this study in accordance with the national legislation and the institutional requirements.

## Author contributions

XG is responsible for the study concept and design. SC, DL, and DJ collected and analyzed the data. RX, JH, ZY, PW, and XX assisted in the analysis and interpretation of data. SC wrote this manuscript. All authors contributed to the article and approved the submitted version.

## Supplementary material

Supplementary Figure 1
**(A–C)** ROC analysis was used to determine the specificity and sensitivity of ERS signatures in the TCGA PRAD cohort. Univariate and multivariate Cox regression analyses were used to validate the prediction of ERS-associated risk signature and other clinical characteristics in GSE70770 **(D, E)** and GSE21034 **(F, G)**.

Supplementary Figure 2 The ERS signature risk grouping and proportions of TME cells in the TCGA PRAD cohort.

Supplementary Figure 3 The relationship between expression of AFP, COL10A1, DNAJB1, EGF, PTGS2, and six tumor-infiltrating lymphocyte.
