# Supplementary figures and images for: Comprehensive analysis of the biological functions of endoplasmic reticulum stress in prostate cancer
Source: Front Endocrinol (Lausanne). 2023 Mar 10;14:1090277. doi: 10.3389/fendo.2023.1090277 (PMC10036859; doi:10.3389/fendo.2023.1090277)

Figure S1

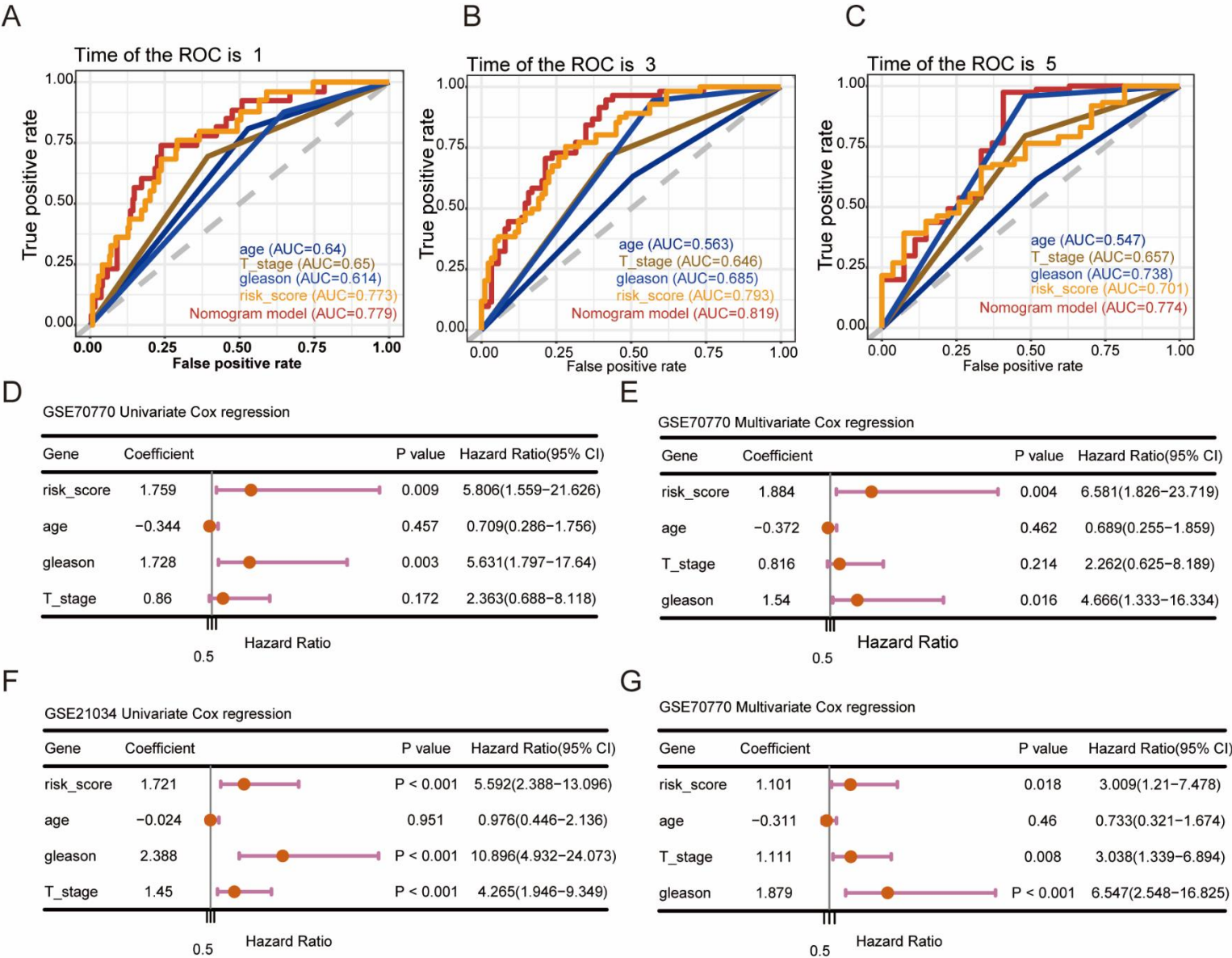

Figure S2

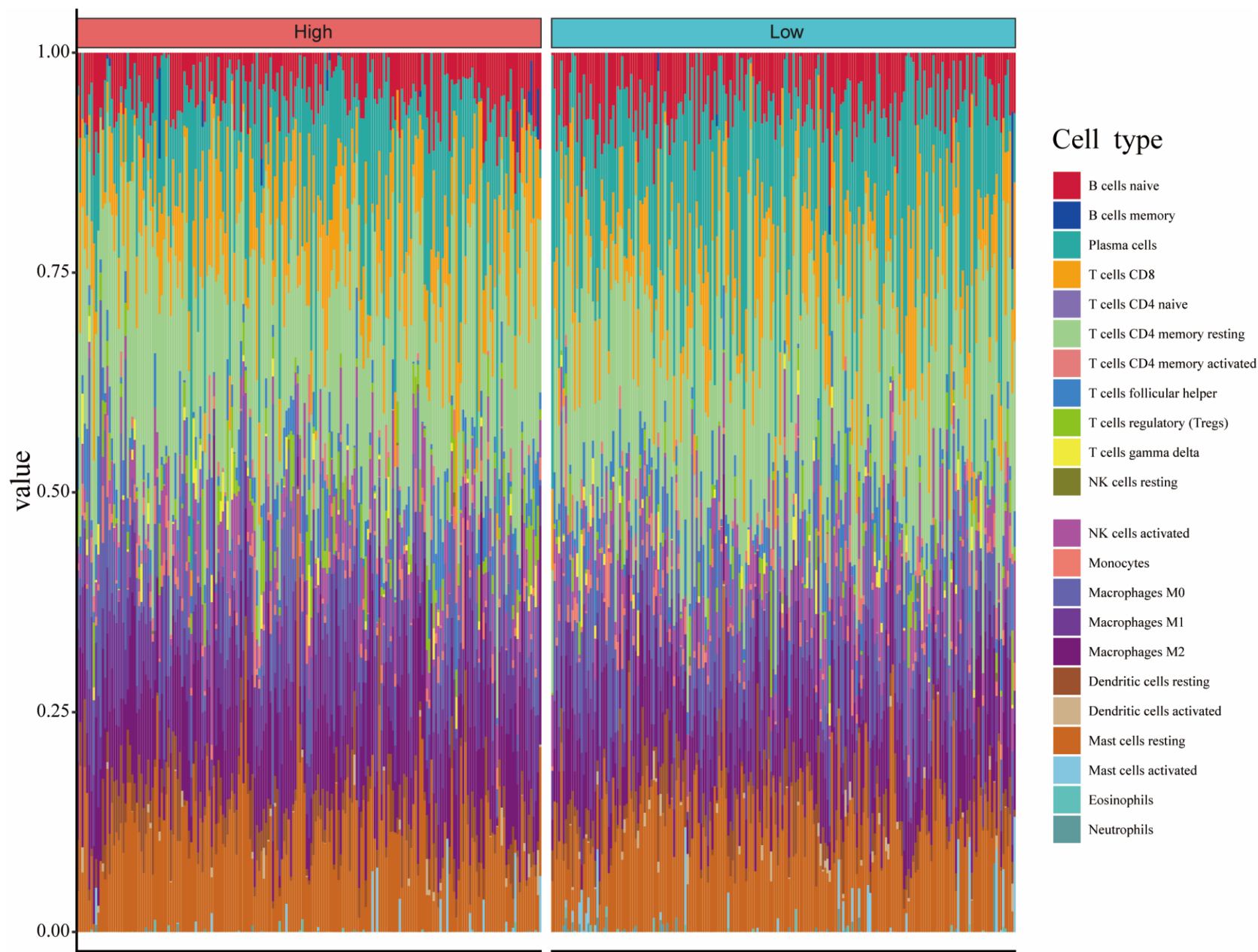

Figure S3

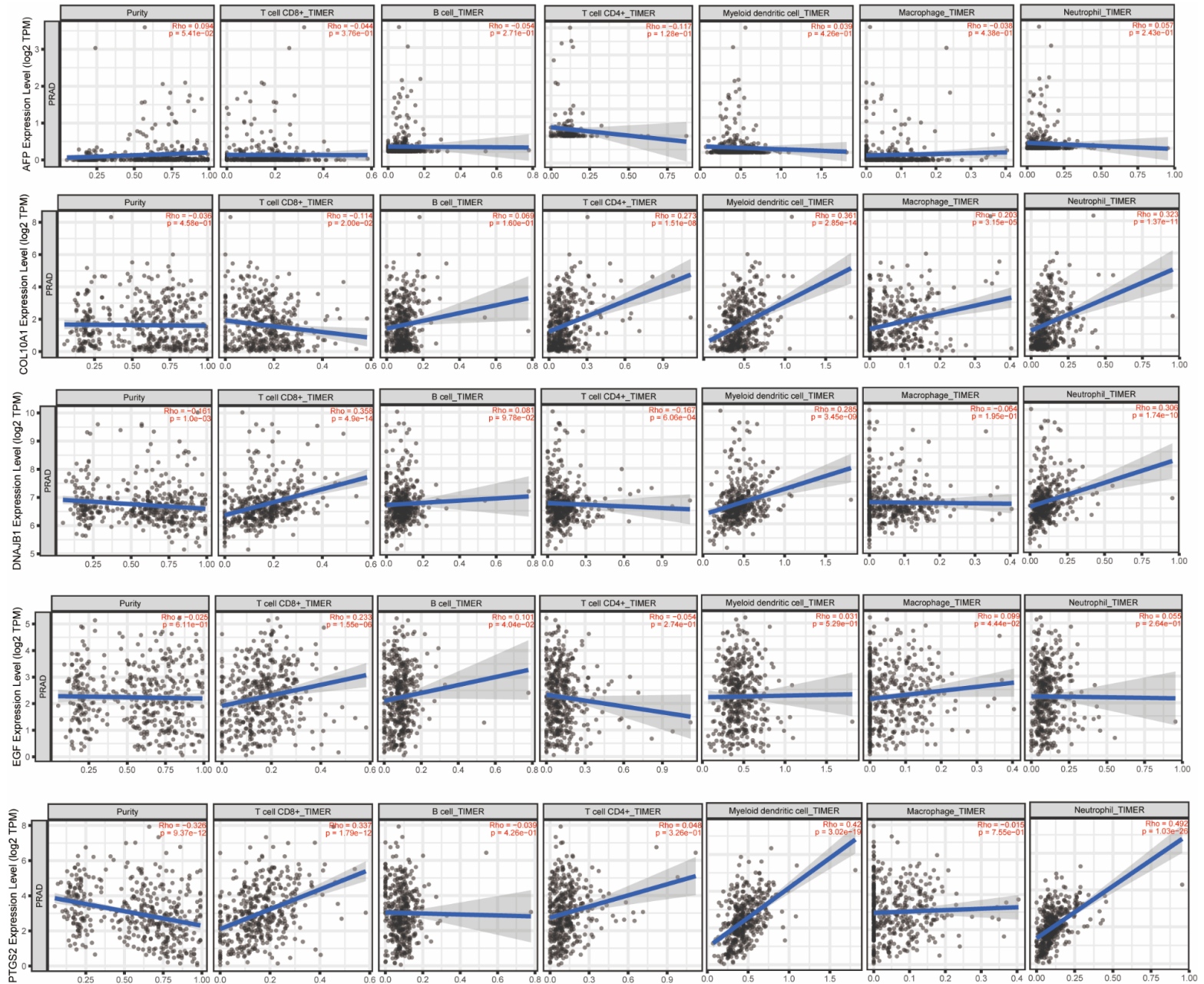

Supplement: Supplementary Figure 1 — (A–C) ROC analysis was used to determine the specificity and sensitivity of ERS signatures in the TCGA PRAD cohort. Univariate and multivariate Cox regression analyses were used to validate the prediction of ERS-associated risk signature and other clinical characteristics in GSE70770 (D, E) and GSE21034 (F, G). [file Image_1.pdf]
