# Supplementary material for: Comprehensive analysis of the biological functions of endoplasmic reticulum stress in prostate cancer
Source: Front Endocrinol (Lausanne). 2023 Mar 10;14:1090277. doi: 10.3389/fendo.2023.1090277 (PMC10036859; doi:10.3389/fendo.2023.1090277)
Supplement: Supplementary file 2 [file Table_1.docx]

**Supplementary Table1**. Clinicopathological characteristics of the TCGA PRAD, GSE70770 and GSE21034 datasets.

| Characteristics | TCGA | GSE70770 | GSE21034 |
| --- | --- | --- | --- |
| Total patients | 411 | 203 | 131 |
| Follow up time Median year(range) | 1.946  (0.077-12.614) | 3.349  (0.030-8.619) | 4.016  (0.115-12.433) |
| Biochemical recurrence status | 411 | 203 | 131 |
| Yes | 45 | 64 | 27 |
| No | 366 | 139 | 104 |
| ERSGs risk score | 411 | 203 | 131 |
| High | 205 | 102 | 66 |
| Low | 206 | 101 | 65 |
| Age |  | 111 | 131 |
| <=60 year | 180 | 48 | 82 |
| >60 year | 226 | 63 | 49 |
| Pathological T stage | 406 | 111 | 131 |
| T1&T2 | 150 | 34 | 85 |
| T3&T4 | 256 | 77 | 46 |
| Gleason score | 406 | 111 | 131 |
| <=7 | 239 | 102 | 116 |
| >7 | 167 | 9 | 15 |
